# Comparative Evaluation of Single-Voxel and Multi-Voxel Proton Magnetic Resonance Spectroscopy Using Small-Sized Voxels in Healthy Beagle Dogs: A Pilot Study

**DOI:** 10.3390/vetsci13090864

**Published:** 2026-08-26

**Authors:** Jong Bong Lee, Gunha Hwang, Seokmin Lee, Youngwon Lee, Hojung Choi, Hee Chun Lee, Tae Sung Hwang

**Affiliations:** 1 Institute of Animal Medicine, College of Veterinary Medicine, Gyeongsang National University, Jinju 52828, Republic of Korea; bong0464@naver.com (J.B.L.); hgh3634@gmail.com (G.H.); 2 College of Veterinary Medicine, Chungnam National University, Daejeon 34134, Republic of Korea; seokmin1992@gmail.com (S.L.); lywon@cnu.ac.kr (Y.L.); hjchoi@cnu.ac.kr (H.C.)

**Keywords:** proton magnetic resonance spectroscopy, single-voxel spectroscopy, multi-voxel spectroscopy, brain chemistry, canine brain

## Abstract

Magnetic resonance imaging has limitations in definitively diagnosing canine brain diseases due to overlapping lesion morphologies. Magnetic resonance spectroscopy (MRS) can complement conventional MR imaging by evaluating brain metabolites at a molecular level, though voxels under 1 cm^3^ have not been routinely evaluated in veterinary medicine. This study evaluated the usefulness of single-voxel spectroscopy (SVS) using small-sized voxels (0.125 cm^3^) by comparing it with multi-voxel spectroscopy (MVS). Both techniques were performed on the white matter and gray matter of the frontoparietal lobe, the caudate nucleus, and the thalamus in seven healthy beagle dogs using a 1.5T system. Brain metabolite concentrations and their ratios to creatine were compared. SVS had a significantly shorter mean acquisition time (4.79 ± 0.29 min) than MVS (11.64 ± 0.23 min). No significant differences were observed in metabolite concentrations or ratios between SVS and MVS following multiplicity correction, confirming no statistically significant discrepancies between the two protocols. No significant differences were found between the left and right hemispheres. While small-voxel SVS is technically feasible and reduces scan time, these findings represent protocol-specific descriptive data in a small sample of healthy dogs and do not establish clinical reference values or diagnostic accuracy.

## Simple Summary

Evaluating brain diseases in dogs often relies on magnetic resonance imaging, but different diseases can show overlapping features on standard scans. Magnetic resonance spectroscopy is a noninvasive complementary tool that evaluates chemical metabolites in brain tissue, offering insights into metabolic status at a molecular level to assist in understanding disease processes. While this method is useful, applying it to the small structures of a canine brain requires using small measurement volumes. This study evaluated a technique called single-voxel spectroscopy, which collects data from one small area at a time, using a voxel size under 1 cubic centimeter. We compared this approach with multi-voxel spectroscopy, which measures multiple areas simultaneously, in seven healthy beagle dogs. The results showed that identifiable brain metabolite peaks were successfully obtained with both single-voxel and multi-voxel methods without statistically significant differences, while the single-voxel method required a significantly shorter scan time. This finding indicates that single-voxel spectroscopy using small volumes is technically feasible in healthy dogs and offers a time-efficient acquisition protocol for future clinical investigations.

## 1. Introduction

Magnetic resonance imaging (MRI) is unsurpassed as a method for evaluating the central nervous system and the brain [1,2]. However, MRI has limitations in achieving a definitive diagnosis due to overlapping lesion morphologies and imaging characteristics among various canine brain diseases [3]. In human medicine, magnetic resonance spectroscopy (MRS) is widely utilized as a complementary tool to MR imaging, especially in certain disease processes [4,5,6]. As a noninvasive method, MRS provides valuable information regarding the chemical composition of living tissue [7]. In humans, it has been successfully applied to help to differentiate brain lesions—including primary brain tumors, inflammatory/infectious diseases, ischemic stroke, and metabolic encephalopathies—as well as to distinguish tumor recurrence from radiation necrosis [4,5,6]. In veterinary medicine, major indications for proton MRS include characterization of intracranial neoplasms, inflammatory central nervous system disorders, ischemic stroke, and metabolic encephalopathies [3,7,8,9].

Protons (^1^H) are the most commonly targeted nuclei in MRS due to their high magnetic sensitivity and natural abundance in organic structures compared to other nuclei such as carbon, sodium, phosphorus, fluorine, and lithium [6]. The primary brain metabolites detected via the ^1^H-MRS technique include N-acetyl aspartate (NAA), choline (Cho), creatine (Cr), myoinositol (mI), lactate (Lac), and glutamate (Glu). Specifically, NAA serves as a neuronal marker present exclusively in neurons, while Cho reflects cell membrane synthesis and degradation. Cr indicates energy metabolism and is generally considered the most stable reference metabolite. Additionally, mI is a pentose sugar, Lac is the end-product of anaerobic glycolysis, and Glu acts as an excitatory neurotransmitter [6,10]. Although the precise quantification of Glu is often challenging at standard clinical field strengths due to spectral overlap with glutamine, evaluating its changes remains highly valuable for assessing neurodegeneration and metabolic shifts [7,8].

MRS can be performed using either single-voxel (data acquired from a single voxel) or multi-voxel techniques (data acquired from multiple voxels simultaneously) [11]. Single-voxel spectroscopy (SVS) typically offers lower spatial resolution and a lower signal-to-noise ratio (SNR) than multi-voxel spectroscopy (MVS), but features significantly shorter acquisition times. In human medicine, due to SNR and spatial resolution constraints, the recommended standard voxel size for the brain is 2 × 2 × 2 cm, with a minimal recommended size of 1 cm^3^ [5,12,13]. However, SVS is less prone to volume contamination because voxel positioning can be precisely manipulated [13]. Conversely, while MVS provides a better SNR and higher spatial resolution, it suffers from longer acquisition times and a higher risk of voxel contamination. This contamination is particularly problematic in the relatively small anatomical structures of the canine brain, where precise voxel positioning remains challenging and volume averaging with adjacent tissues or cerebrospinal fluid easily occurs [12,14].

Given the prolonged scan times required for MVS under clinical anesthesia, SVS is often more suitable for small, localized lesions [15]. In veterinary medicine, only a limited number of studies have measured brain metabolite concentrations using SVS [7,8,16,17]. Because the canine brain contains much smaller distinct anatomical regions than the human brain, there is an evident need to investigate SVS protocols utilizing smaller voxel sizes. To the best of our knowledge, no veterinary studies evaluating voxel sizes under 1 cm^3^ have been reported.

Therefore, the primary purpose of this prospective pilot feasibility study was to evaluate the technical feasibility and operational characteristics of SVS using small-sized voxels (0.125 cm^3^) compared to MVS in seven healthy Beagle dogs. We hypothesized that small-voxel SVS could be acquired in healthy canine brains with significantly shorter acquisition times without statistically significant procedural discrepancies in primary stable metabolite ratios when compared to MVS under a standardized 1.5 T protocol.

## 2. Materials and Methods

### 2.1. Experimental Animals

Seven healthy beagle dogs (mean weight: 10.3 kg, range: 8.9–12.7 kg; mean age: 5 years; seven castrated males) were enrolled in this study. Prior to the study, all dogs underwent comprehensive physical and neurological examinations, thoracic radiography, abdominal ultrasonography, echocardiography, conventional brain magnetic resonance imaging (MRI), and cerebrospinal fluid (CSF) analysis to confirm their healthy status. The dogs were fasted for approximately 12 h before the magnetic resonance spectroscopy (MRS) examinations. Anesthesia was induced with an intravenous injection of alfaxalone (2 mg/kg, IV; Alfaxan; Careside Co., Ltd., Seongnam, Republic of Korea) and maintained with isoflurane (Irfan; Hana Pharm Co., Ltd., Hwaseong, Republic of Korea) in oxygen (2.0 L/min) via endotracheal intubation. Electrocardiography (ECG), oxygen saturation, and respiratory rate were continuously monitored during all procedures. All experimental protocols and animal care procedures were approved by the Institutional Animal Care and Use Committee (IACUC) of Gyeongsang National University (Approval No. GNU-221230-D0197).

### 2.2. Conventional MRI Imaging

Brain MRI and MRS acquisitions for all dogs were performed using a 1.5 T magnet scanner (Vantage Elan™; Canon Medical Systems, Otawara, Tochigi, Japan) with a medium 16-channel flex SPEEDER coil. All dogs were scanned in sternal recumbency. Conventional MRI was performed to accurately guide and select voxel locations during MRS imaging. The conventional MRI protocol consisted of transverse (TR/TE: 6141/108 ms), sagittal (TR/TE: 4071/84 ms), and dorsal (TR/TE: 3460/84 ms) T2-weighted sequences of the brain. The primary scanning parameters included a slice thickness of 3.0 mm and a field of view of 120 × 120 mm.

### 2.3. Magnetic Resonance Spectroscopy

**Figure 1:**
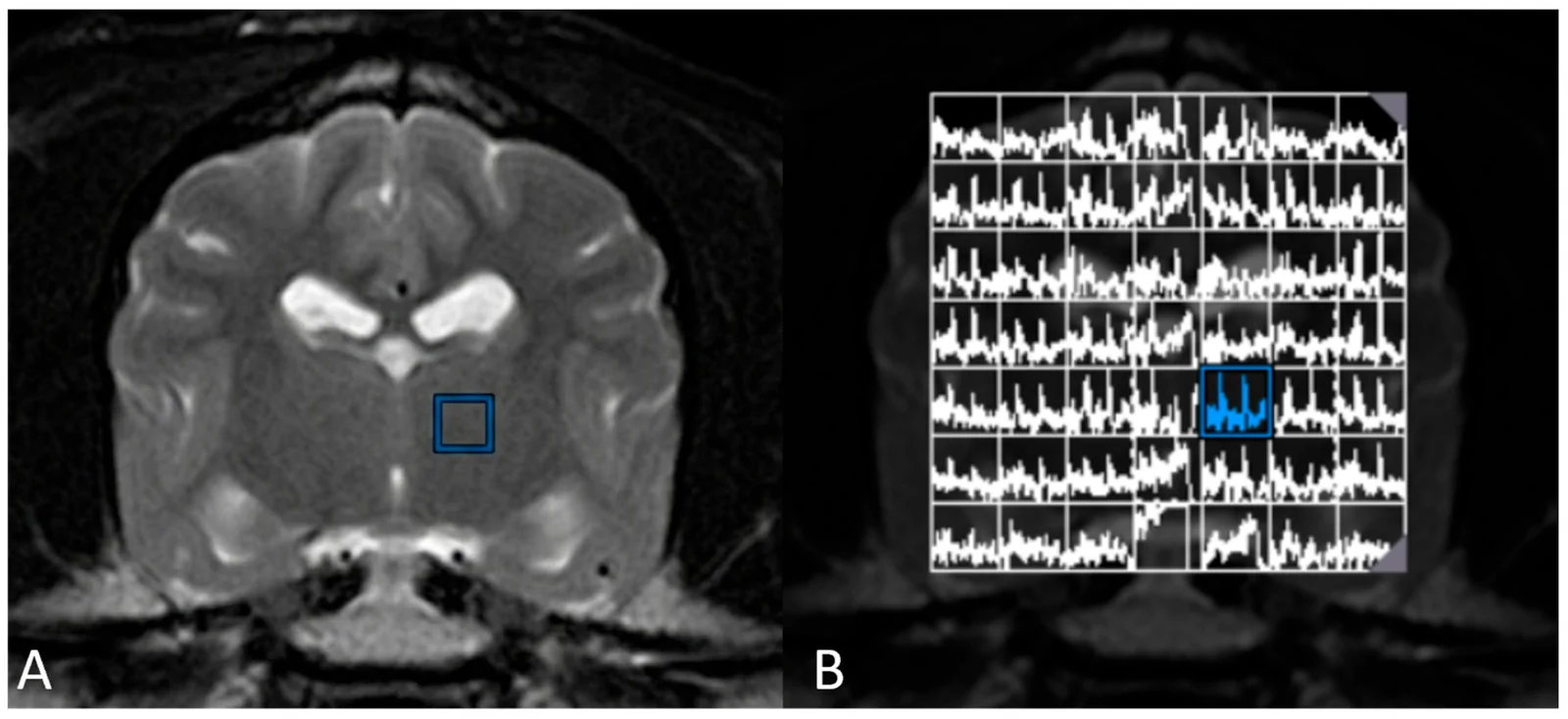
Representative transverse T2-weighted MR images comparing region of interest placements within the thalamus of a healthy Beagle dog (*n* = 7). (**A**) Single-voxel spectroscopy placement (the blue box indicates the targeted single-voxel ROI). (**B**) Multi-voxel spectroscopy placement (the blue highlighted area indicates the selected voxel within the multi-voxel grid).

Before conducting MRS, localized shimming was performed to optimize magnetic field homogeneity, maintaining the water peak full width at half maximum under 15 Hz for all acquisitions. Single-voxel spectroscopy (SVS; TR/TE: 2000/136 ms, flip angle: 90°, voxel size: 0.5 × 0.5 × 0.5 cm, point-resolved spectroscopy [PRESS]) and multi-voxel spectroscopy (MVS; TR/TE: 1500/136 ms, flip angle: 90°, volume of interest [VOI]: 8 × 8 × 1, individual voxel size: 0.5 × 0.5 × 0.5 cm, PRESS) were performed on all dogs. Based on established anatomical landmarks on conventional T2-weighted images, the targeted voxel locations were consistently placed in both the left and right hemispheres of the white matter (WM) and gray matter (GM) of the frontoparietal lobe individually, the caudate nucleus, and the thalamus (Figure 1). For SVS, careful positioning was performed to minimize volume contamination from adjacent brain lobes, bony structures, and CSF. For MVS, the grid was aligned to achieve maximum symmetry between the left and right hemispheres.

Metabolite concentrations were quantified at the respective peak areas of the spectra: N-acetyl aspartate (NAA) at 2.01 ppm, choline (Cho) at 3.2 ppm, creatine (Cr) at 3.0 ppm, and glutamate (Glu) at 2.25 ppm. All spectral processing, curve fitting, and metabolite quantification were performed using the manufacturer’s proprietary software interface on the MRI console. In this study, we analyzed the absolute concentrations of NAA, Cho, Cr, and Glu, as well as the NAA/Cr and Cho/Cr ratios, which are widely utilized clinical markers in ^1^H-MRS.

### 2.4. Statistical Analysis

Statistical analyses were conducted using SPSS software (Version 27.0; IBM Corp., Armonk, NY, USA). The Shapiro–Wilk test was applied to assess data normality, revealing non-normal distributions across the dataset; therefore, non-parametric methods were strictly employed. The Wilcoxon signed-rank test was used to compare metabolite concentrations and ratios between SVS and MVS techniques, as well as to evaluate interhemispheric differences (left versus right). To stringently control for Type I errors arising from multiple comparisons (24 metabolite-by-region interactions), the Benjamini–Hochberg false discovery rate (FDR) procedure was applied to the initial unadjusted *p*-values. The effect size (*r*) for the Wilcoxon tests was calculated using the formula *r* = |Z|/√N. Differences among the four distinct brain regions were analyzed using the Kruskal–Wallis test, followed by Dunn’s post hoc test with Bonferroni correction. Continuous variables are reported as the mean ± standard deviation. Statistical significance was defined as an adjusted p-value (*p*_adj_) of <0.05.

Google Gemini (version: Gemini 1.5 Pro; Google LLC, Mountain View, CA, USA) was used for English language editing during manuscript preparation.

## 3. Results

Among the acquired data, four spectra with poor quality were excluded from the final analysis. In SVS, one spectrum from the thalamus voxel was excluded due to an unstable baseline. In MVS, three spectra were excluded due to lipid contamination and peak identification difficulties: one from the frontoparietal white matter (one dog) and two from the caudate nucleus (two dogs). The mean acquisition time was significantly shorter for SVS (4.79 ± 0.29 min) than for MVS (11.64 ± 0.23 min).

**Figure 2:**
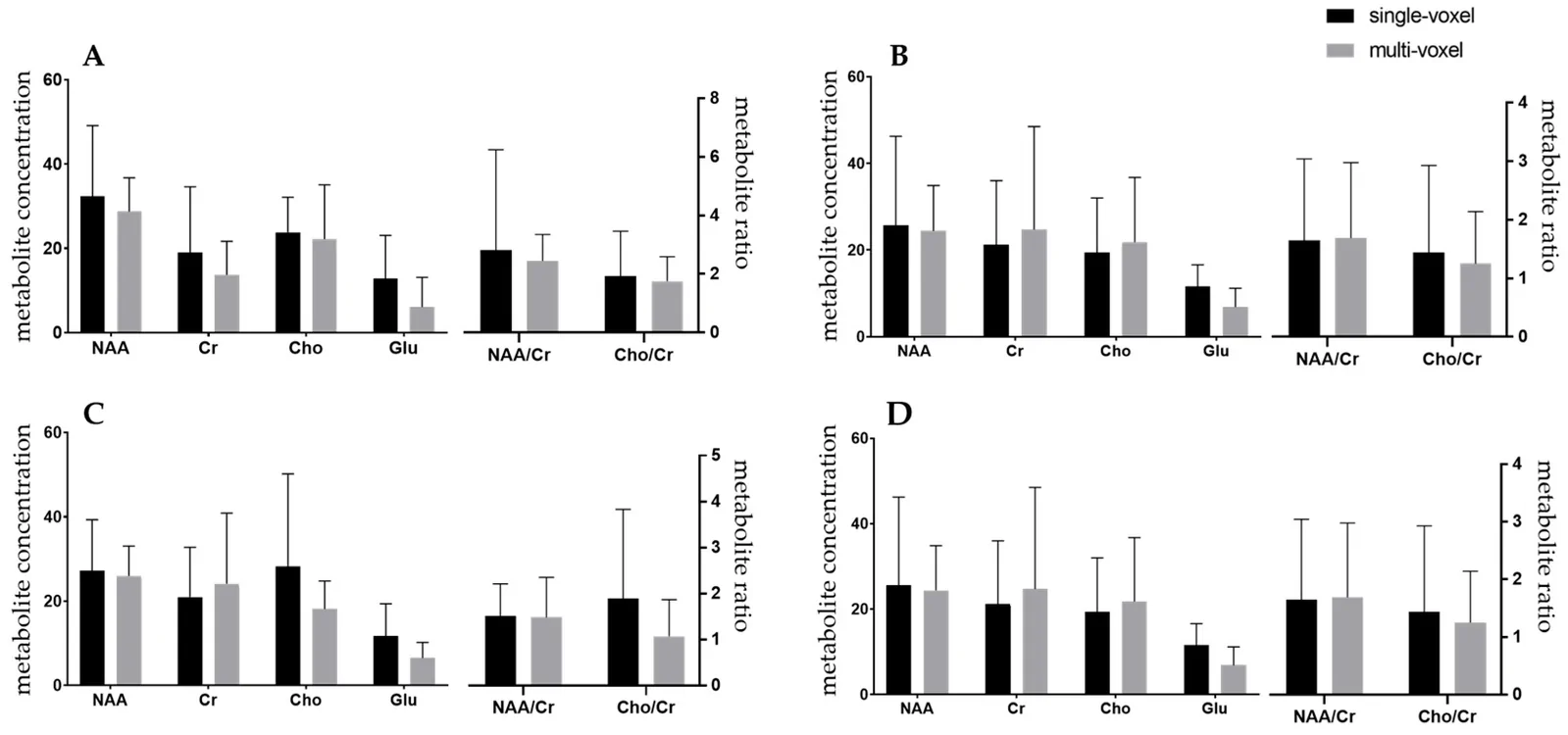
Comparison of brain metabolite concentrations (NAA, Cr, Cho, and Glu) and metabolite ratios relative to creatine (NAA/Cr and Cho/Cr) between single-voxel spectroscopy (SVS) and multi-voxel spectroscopy (MVS) across four evaluated brain regions ((**A**). WM of frontoparietal lobe, (**B**). GM of frontoparietal lobe, (**C**). Caudate nucleus, and (**D**). Thalamus). Data are presented as mean ± standard deviation. Following Benjamini–Hochberg false discovery rate correction, no statistically significant differences were observed between SVS and MVS for any metabolite or ratio (*p*_adj_ > 0.05); NAA = N-acetyl aspartate; Cr = creatine; Cho = choline; Glu = glutamate.

The mean metabolite concentrations and their ratios relative to creatine (Cr) across the evaluated brain regions are presented in Figure 2, with detailed numerical data provided in Supplementary Table S1. In the initial unadjusted paired analysis comparing SVS and MVS, nominal differences were noted in glutamate (Glu) concentrations within the gray matter of the frontoparietal lobe (*p* = 0.026, *r* = 0.42), the caudate nucleus (*p* = 0.041, *r* = 0.38), and the thalamus (*p* = 0.019, *r* = 0.45). However, following the application of the Benjamini–Hochberg FDR correction across all 24 metabolite-by-region comparisons, none of these differences retained statistical significance (gray matter of the frontoparietal lobe, *p*_adj_ = 0.312; caudate nucleus, *p*_adj_ = 0.328; thalamus, *p*_adj_ = 0.312). Consequently, no statistically significant disparities in the primary metabolite profiles (NAA, Cr, Cho, Glu, NAA/Cr, and Cho/Cr) were confirmed between the two spectroscopic techniques.

**Figure 3:**
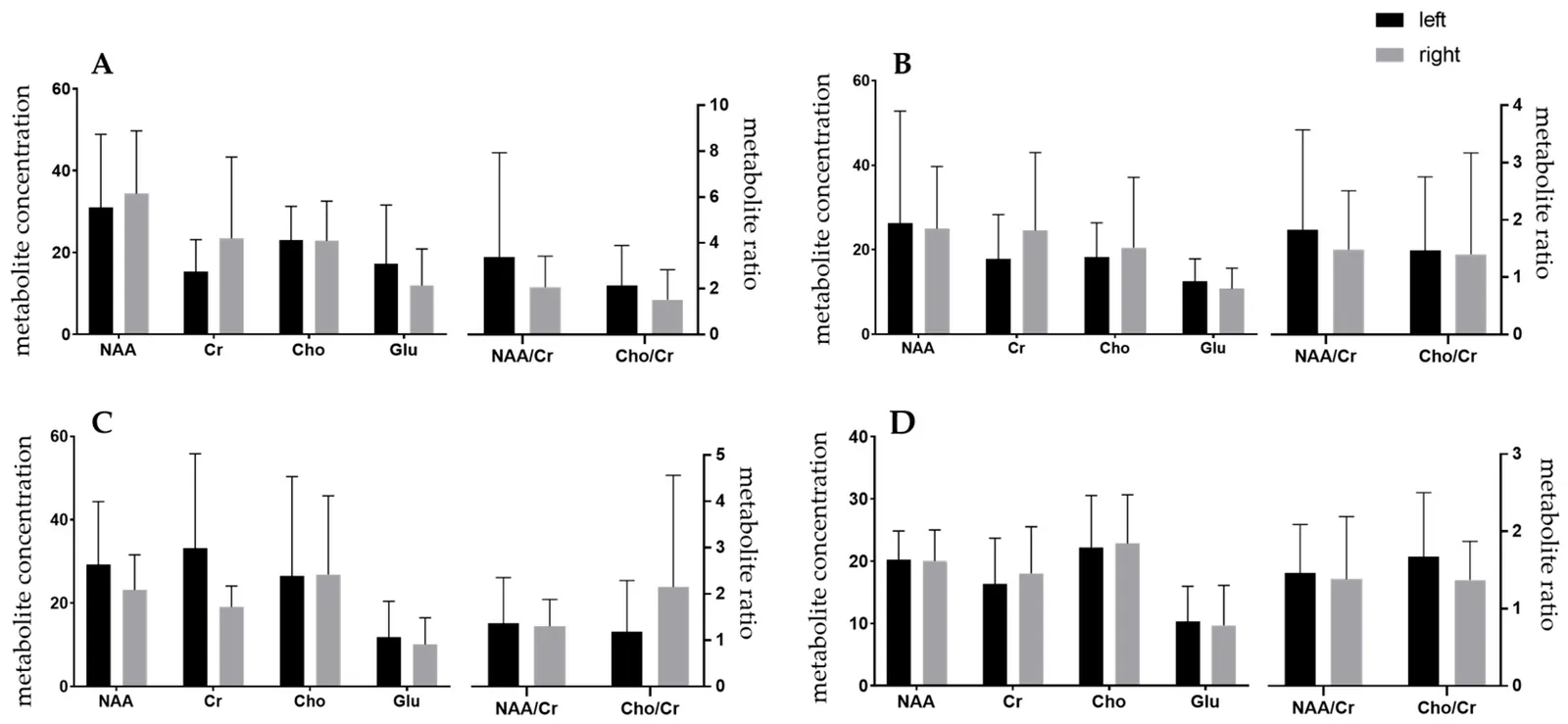
Comparison of brain metabolite concentrations and ratios between the left and right hemispheres in single-voxel spectroscopy across four evaluated brain regions ((**A**). WM of frontoparietal lobe, (**B**). GM of frontoparietal lobe, (**C**). Caudate nucleus, and (**D**). Thalamus). Data are presented as mean ± standard deviation. No statistically significant interhemispheric asymmetries were observed (*p* > 0.05). FP = frontoparietal; WM = white matter; GM = gray matter; NAA = N-acetyl aspartate; Cr = creatine; Cho = choline; Glu = glutamate.

**Figure 4:**
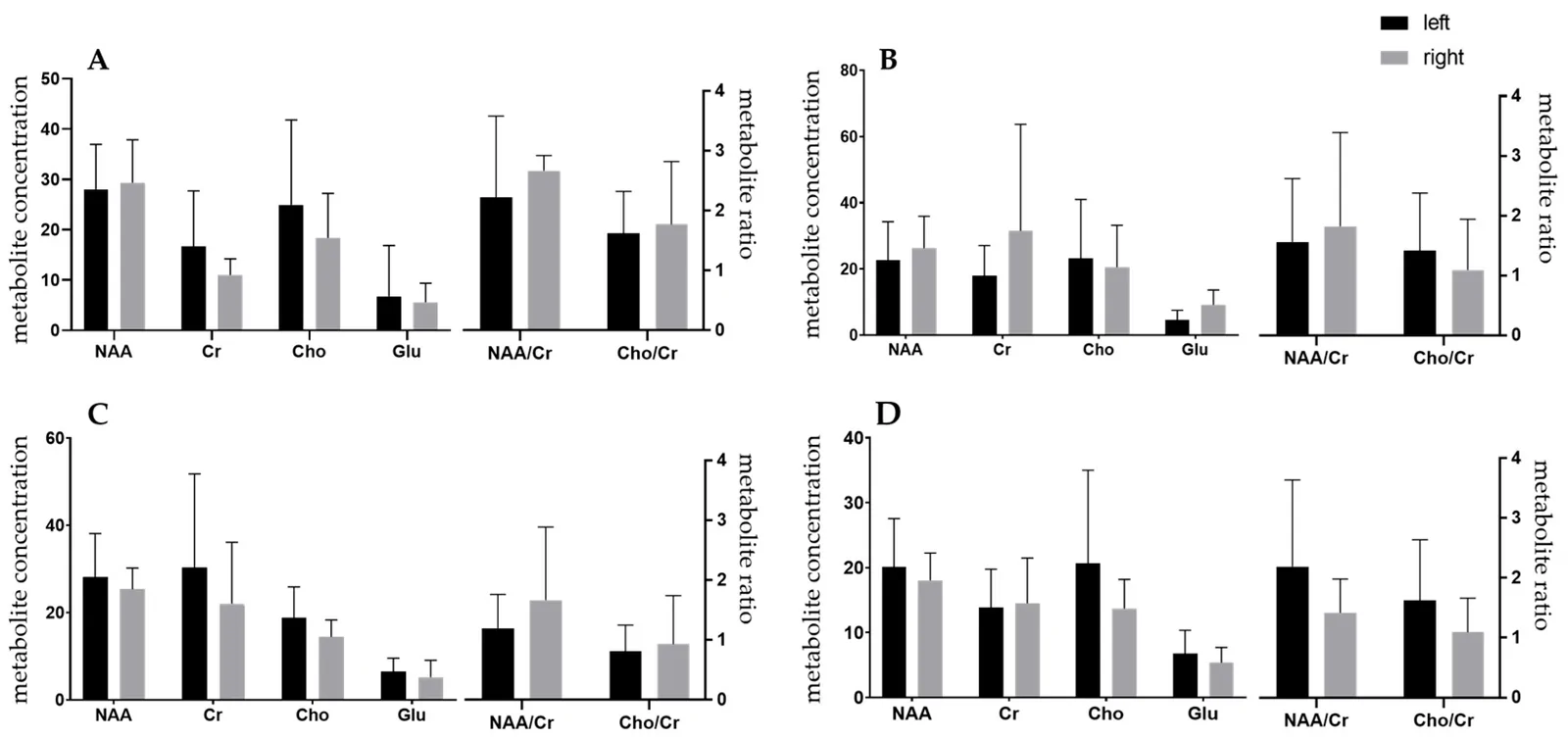
Comparison of brain metabolite concentrations and ratios between the left and right hemispheres in multi-voxel spectroscopy across four evaluated brain regions ((**A**). WM of frontoparietal lobe, (**B**). GM of frontoparietal lobe, (**C**). Caudate nucleus, and (**D**). Thalamus). Data are presented as mean ± standard deviation. No statistically significant interhemispheric asymmetries were observed (*p* > 0.05). FP = frontoparietal; WM = white matter; GM = gray matter; NAA = N-acetyl aspartate; Cr = creatine; Cho = choline; Glu = glutamate.

Data comparing the left and right hemispheres are illustrated in Figure 3 and Figure 4, with comprehensive numerical values detailed in Supplementary Tables S2 and S3. For both SVS and MVS, no statistically significant interhemispheric asymmetries were detected for any of the analyzed metabolites or ratios. Furthermore, regional comparisons among the frontoparietal white matter, frontoparietal gray matter, caudate nucleus, and thalamus yielded no significant differences in metabolite profiles in this study.

## 4. Discussion

Only a limited number of studies have compared SVS and MVS in veterinary medicine, and none have investigated the application of small-sized voxels (<1 cm^3^) in SVS [15]. The present study was designed to address this gap. Our findings demonstrated no significant differences in major metabolite concentrations and metabolite ratios between SVS and MVS, which is consistent with previous literature [15]. However, it is worth noting that our experimental setup differed from prior studies regarding magnetic field strength, targeted brain regions, evaluated metabolites, and the smaller voxel size utilized in SVS [15].

In line with past reports, we observed no statistically significant differences in major metabolite concentrations or ratios between SVS and MVS following Benjamini–Hochberg FDR correction. Although initial unadjusted analyses indicated nominal differences in Glu levels across certain brain regions, these did not remain statistically significant after controlling for multiple testing. Nevertheless, the identification and quantification of metabolites in proton MRS depend heavily on the echo time (TE) [6,10]. A longer TE (e.g., 136 ms) facilitates the clear evaluation of metabolites with simpler coupling patterns and longer T2 relaxation times, such as NAA, Cho, Cr, and Lac [10,12]. Conversely, a shorter TE is highly recommended to identify strongly coupled resonances, including Glu, Gln, glucose, macromolecular proteins, and lipids [10,12]. Furthermore, higher magnetic field strengths significantly improve spectral resolution for these strongly coupled metabolites [10,18]. In this study, the nominal variance observed in Glu concentrations prior to correction likely reflects the limited spectral resolution under a 1.5 T field strength combined with a long TE (136 ms), which makes the distinct separation of Glu from Gln technically challenging [10,12]. Consequently, further studies employing a higher field strength (3.0 T or higher) and a short TE protocol are warranted to evaluate glutamate concentrations more accurately.

The brain metabolites routinely detected via ^1^H-MRS include NAA, Cho, Cr, mI, Lac, Glu, Gln, lipids, and alanine [6,10]. Among these, NAA, Cho, and Cr are the primary biomarkers used to diagnose human neurological disorders [5]. In veterinary medicine, Glu has additionally been evaluated alongside these primary metabolites to assist in clinical diagnosis [7,8,9,19]. Accordingly, we measured and compared the levels of NAA, Cho, Cr, and Glu. Due to inter-subject variations in tissue susceptibility, coil loading, and receiver gain, metabolite-to-creatine ratios are more commonly reported than absolute concentrations [20,21]. Ratios have the advantage of being largely unaffected by CSF contamination within the voxel, as metabolite concentrations in CSF are negligible [21,22]. However, these ratios are inherently sensitive to concentration changes in both the numerator and the denominator [21,22]. Because Cr is generally considered the most stable metabolite under normal physiological conditions, it is widely used as the reference standard [6]. In the current study, we utilized both absolute metabolite concentrations and Cr-normalized ratios to ensure a robust evaluation.

While MVS offers a superior signal-to-noise ratio (SNR) and spatial coverage [12,13], it has notable disadvantages, including longer scan times and susceptibility to voxel contamination from adjacent non-brain structures, such as bone, air, and adipose tissue [13]. Conversely, SVS requires a voxel size large enough to yield adequate SNR and diagnostic-quality spectra. However, larger SVS voxels increase the risk of volume averaging, incorporating adjacent brain lobes or CSF, which can distort the resulting spectrum [14,15]. In our study, SVS using a 0.5 × 0.5 × 0.5 cm voxel (0.125 cm^3^) exhibited a minor decrease in SNR but still provided clearly identifiable metabolite peaks, with no statistically significant differences detected relative to MVS in this exploratory sample. Achieving high magnetic field homogeneity is critical for high-quality ^1^H-MRS. Small voxel volumes inherently contain fewer spatial field gradients, making localized *B*_0_ shimming substantially easier and more effective [23]. Furthermore, SVS is particularly advantageous when evaluating well-defined focal lesions, minimizing partial volume averaging from surrounding non-lesional tissues [24].

Evaluating spectral quality is critical to ensure diagnostic reliability. In a previous study, spectrum quality was assessed using a 5-point scale, where a score of 3 represented marginal quality and scores of 1 or 2 were classified as non-diagnostic [14]. In our study, non-diagnostic spectra (scores of 1 and 2) were excluded. One SVS spectrum from the thalamus was excluded due to severe baseline instability, which is a known limitation linked to the lower intrinsic SNR of SVS. In MVS, three spectra (from the frontoparietal white matter and caudate nucleus) were excluded due to prominent lipid peaks that obscured other metabolite peaks. This contamination was likely due to the inherent limitation of the fixed multi-voxel grid, which increases the susceptibility to partial volume effects along the anatomical borders of the small canine brain. These findings emphasize that spectral quality control is as crucial as the metabolite measurements themselves.

In human medicine, no significant differences in brain metabolite concentrations have been reported between the left and right hemispheres [22,25]. In veterinary medicine, hemisphere comparison studies have yielded conflicting results; two studies reported no hemispheric asymmetry, while one reported significant differences [16,17,26]. In the present study, no significant differences were observed between the left and right hemispheres in either SVS or MVS. Additionally, as noted by Lee et al. [15], data from the contralateral normal brain can serve as a valuable internal control for individual dogs with brain lesions, mitigating inter-subject biological variability. This bilateral symmetry may be attributed to the minimal voxel contamination achieved through our ultra-small voxel placement. Although previous literature indicates that Cho concentrations are significantly higher in the thalamus than in other brain regions [15,17,26,27], we did not observe significant regional differences in Cho levels. This lack of statistical significance among brain regions was likely a result of the relatively small sample size of our cohort. Additionally, unlike previous studies that utilized larger voxels susceptible to volume averaging with surrounding tissues, our ultra-small voxel (0.125 cm^3^) precisely targeted the core thalamic parenchyma, which may also account for the differences in regional metabolite profiles.

This study has several inherent limitations. First, the evaluation was conducted on a small, homogeneous sample (n=7 healthy castrated male Beagles) using a single 1.5 T scanner and proprietary software. Second, failure to detect statistically significant differences between SVS and MVS in this small exploratory sample does not demonstrate equivalence or agreement between the two techniques, as statistical power was limited by sample size and necessary spectral exclusions.

## 5. Conclusions

Small-voxel SVS (0.125 cm^3^) was successfully implemented in seven healthy Beagle dogs, offering a technically feasible approach with substantially reduced acquisition time compared to MVS. Within this small exploratory sample, no statistically significant differences were detected in major stable metabolite profiles between the two protocols. However, these findings represent preliminary, protocol-specific descriptive data. Future studies involving larger cohorts, higher magnetic field strengths, and dogs with confirmed intracranial pathologies are needed to evaluate the clinical diagnostic utility of small-voxel SVS in veterinary neurology.

## Data Availability

The raw data supporting the conclusions of this article will be made available by the authors on request.

## Supplementary Materials

The following supporting information can be downloaded at: https://www.mdpi.com/article/10.3390/vetsci13090864/s1, Table S1: Comparison of metabolite concentration and metabolite ratio in the brain regions between single-voxel and multi-voxel spectroscopy (*n* = 7); Table S2: Comparison of brain metabolite concentrations and ratios between the left and right hemispheres in single-voxel spectroscopy (*n* = 7); Table S3: Comparison of brain metabolite concentrations and ratios between the left and right hemispheres in multi-voxel spectroscopy (*n* = 7); Table S4: Individual measurements of brain metabolite concentrations and ratios in single-voxel spectroscopy across four evaluated brain regions (*n* = 7); Table S5: Individual measurements of brain metabolite concentrations and ratios in multi-voxel spectroscopy across four evaluated brain regions (*n* = 7).

## Abbreviations

The following abbreviations are used in this manuscript:

MRI	Magnetic resonance imaging
MRS	Magnetic resonance spectroscopy
SVS	Single-voxel spectroscopy
MVS	Multi-voxel spectroscopy
PRESS	Point-resolved spectroscopy
VOI	Volume of interest
SNR	Signal-to-noise ratio
TR	Repetition time
TE	Echo time
NAA	N-acetyl aspartate
Cho	Choline
Cr	Creatine
mI	Myoinositol
Lac	Lactate
Glu	Glutamate
Gln	Glutamine
WM	White matter
GM	Gray matter
CSF	Cerebrospinal fluid
CNS	Central nervous system
IACUC	Institutional Animal Care and Use Committee
SD	Standard deviation
ppm	Parts per million
