# Supplementary material for: Comparative Evaluation of Single-Voxel and Multi-Voxel Proton Magnetic Resonance Spectroscopy Using Small-Sized Voxels in Healthy Beagle Dogs: A Pilot Study
Source: Vet Sci. 2026 Aug 26;13(9):864. doi: 10.3390/vetsci13090864 (PMC13611929; doi:10.3390/vetsci13090864)
Supplement: Supplementary file 1 [file vetsci-13-00864-s001.zip › vetsci-4471009-supplementary.pdf]

veterinary sciences

MDPI

Supplementary Table S1. Comparison of metabolite concentration and metabolite ratio in the brain regions between single-voxel and multi-voxel spectroscopy (n = 7).

	WM of frontoparietal lobe		GM of frontoparietal lobe		Caudate nucleus		Thalamus	
	SVS	MVS	SVS	MVS	SVS	MVS	SVS	MVS
NAA	32.35 ± 16.72	28.71 ± 8.02	25.64 ± 20.62	24.41 ± 10.49	27.25 ± 12.09	25.98 ± 7.08	21.16 ± 5.79	19.23 ± 6.13
Cr	18.93 ± 15.64	13.68 ± 7.96	21.21 ± 14.81	24.72 ± 23.79	20.96 ± 11.82	24.13 ± 16.76	17.23 ± 6.84	14.58 ± 6.27
Cho	23.79 ± 8.32	22.16 ± 12.88	19.37 ± 12.64	21.85 ± 14.92	28.19 ± 22.03	18.13 ± 6.66	21.60 ± 8.11	16.95 ± 11.23
Glu	12.81 ± 10.25	6.07 ± 7.01	11.62 ± 5.00	6.85 ± 4.35	11.76 ± 7.61	6.49 ± 3.72	10.65 ± 5.99	5.84 ± 3.02
NAA/Cr	2.82 ± 3.42	2.45 ± 0.90	1.65 ± 1.39	1.69 ± 1.29	1.51 ± 0.70	1.48 ± 0.87	1.46 ± 0.68	1.80 ± 1.18
Cho/Cr	1.93 ± 1.53	1.75 ± 0.84	1.44 ± 1.49	1.25 ± 0.89	1.89 ± 1.94	1.07 ± 0.80	1.42 ± 0.69	1.29 ± 0.83

SVS = single-voxel spectroscopy, MVS = multi-voxel spectroscopy, WM = white matter, GM = gray matter, NAA = N-acetyl aspartate, Cr = creatine, Cho = choline, Glu = glutamate.
Metabolite concentration and metabolite ratio are presented as mean ± SD.
Due to spectral quality exclusions, valid paired observations were n = 6 for the SVS thalamus, n = 6 for the MVS frontoparietal white matter, and n = 5 for the MVS caudate nucleus; all other evaluated regions included n = 7 valid spectra.

Supplementary Table S2. Comparison of brain metabolite concentrations and ratios between the left and right hemispheres in single-voxel spectroscopy (n = 7).

	WM of frontoparietal lobe		GM of frontoparietal lobe		Caudate nucleus		Thalamus	
	Left	Right	Left	Right	Left	Right	Left	Right
NAA	30.99 ± 17.88	34.35 ± 15.36	26.28 ± 26.54	25.00 ± 14.69	29.27 ± 15.06	23.16 ± 8.42	20.19 ± 4.66	20.04 ± 4.98
Cr	15.30 ± 7.84	23.45 ± 19.88	17.87 ± 10.45	24.55 ± 18.44	33.16 ± 22.66	19.09 ± 5.02	16.31 ± 7.39	18.05 ± 7.47
Cho	22.94 ± 8.32	22.81 ± 9.73	18.25 ± 8.13	20.48 ± 16.65	26.51 ± 23.84	26.88 ± 18.87	22.14 ± 8.41	22.87 ± 7.81
Glu	17.22 ± 14.37	11.90 ± 8.95	12.49 ± 5.35	10.76 ± 4.87	11.84 ± 8.62	10.11 ± 6.37	10.35 ± 5.62	9.68 ± 6.44
NAA/Cr	3.38 ± 4.56	2.06 ± 1.36	1.82 ± 1.75	1.48 ± 1.03	1.37 ± 0.98	1.30 ± 0.58	1.46 ± 0.63	1.38 ± 0.81
Cho/Cr	2.14 ± 1.75	1.51 ± 1.32	1.47 ± 1.28	1.40 ± 1.77	1.18 ± 1.11	2.15 ± 2.41	1.67 ± 0.83	1.37 ± 0.50

WM = white matter, GM = gray matter, NAA = N-acetyl aspartate, Cr = creatine, Cho = choline, Glu = glutamate.
Metabolite concentration and metabolite ratio are presented as mean ± SD.
Due to spectral quality exclusions, valid paired observations were n = 6 for the SVS thalamus, n = 6 for the MVS frontoparietal white matter, and n = 5 for the MVS caudate nucleus; all other evaluated regions included n = 7 valid spectra.

Supplementary Table S3. Comparison of brain metabolite concentrations and ratios between the left and right hemispheres in multi-voxel spectroscopy (n = 7).

	WM of frontoparietal lobe		GM of frontoparietal lobe		Caudate nucleus		Thalamus	
	Left	Right	Left	Right	Left	Right	Left	Right
NAA	28.00 ± 8.95	29.31 ± 8.55	22.57 ± 11.70	26.26 ± 9.67	28.16 ± 9.99	25.39 ± 4.83	20.07 ± 7.48	18.03 ± 4.22
Cr	16.67 ± 11.03	10.98 ± 3.21	17.94 ± 9.14	31.51 ± 32.18	30.27 ± 21.55	22.03 ± 14.10	13.87 ± 5.89	14.50 ± 6.97
Cho	24.90 ± 16.89	18.36 ± 8.84	23.20 ± 17.80	20.50 ± 12.71	18.79 ± 7.09	14.45 ± 3.88	20.68 ± 14.32	13.68 ± 4.51
Glu	6.72 ± 10.13	5.54 ± 3.81	4.56 ± 2.95	9.14 ± 4.49	6.53 ± 3.02	5.18 ± 3.89	6.75 ± 3.59	5.35 ± 2.36
NAA/Cr	2.22 ± 1.36	2.67 ± 0.25	1.56 ± 1.06	1.82 ± 1.57	1.20 ± 0.56	1.67 ± 1.22	2.19 ± 1.45	1.42 ± 0.56
Cho/Cr	1.62 ± 0.70	1.77 ± 1.05	1.42 ± 0.96	1.09 ± 0.85	0.81 ± 0.44	0.93 ± 0.81	1.63 ± 1.01	1.09 ± 0.57

WM = white matter, GM = gray matter, NAA = N-acetyl aspartate, Cr = creatine, Cho = choline, Glu = glutamate.
Metabolite concentration and metabolite ratio are presented as mean ± SD.
Due to spectral quality exclusions, valid paired observations were n = 6 for the SVS thalamus, n = 6 for the MVS frontoparietal white matter, and n = 5 for the MVS caudate nucleus; all other evaluated regions included n = 7 valid spectra.

Supplementary Table S4. Individual measurements of brain metabolite concentrations and ratios in single-voxel spectroscopy across four evaluated brain regions (n = 7).

	WM of frontoparietal lobe		GM of frontoparietal lobe		Caudate nucleus		Thalamus	
	Left	Right	Left	Right	Left	Right	Left	Right
NAA								
1	19.000	55.050	22.240	18.200	31.010	25.580	-	-
2	19.810	33.130	10.790	36.950	59.050	23.590	19.400	21.940
3	36.780	42.300	85.900	10.950	28.920	22.450	23.230	23.480
4	67.470	35.100	17.150	7.450	21.870	25.100	26.600	15.080
5	35.110	19.150	16.870	38.200	30.440	37.300	18.880	24.750
6	19.670	10.450	12.930	18.640	24.050	19.080	20.380	22.490
7	19.100	45.320	18.100	44.660	9.550	9.060	12.690	12.540
Cr								
1	15.900	12.520	8.340	12.380	14.920	23.160	-	-
2	13.910	26.870	6.830	11.540	52.020	17.710	22.800	12.240
3	25.240	16.250	15.800	19.370	31.340	12.680	22.240	16.150
4	4.960	13.830	29.050	58.000	11.560	12.290	19.110	25.260
5	16.470	7.630	14.580	17.710	65.760	21.520	7.950	18.410
6	6.380	20.770	15.420	10.240	8.090	24.960	19.770	8.390
7	24.270	66.340	35.100	42.650	48.450	21.350	6.010	27.870
Cho								
1	29.170	14.720	20.040	32.390	1.050	44.920	-	-
2	33.650	16.420	27.020	8.950	26.250	11.520	16.790	13.840
3	10.960	28.060	18.820	11.050	76.440	2.980	21.790	17.940
4	28.370	10.590	29.570	24.550	29.180	16.900	37.300	22.640
5	16.770	32.450	14.080	3.450	14.370	42.820	13.570	33.610
6	16.250	36.540	7.570	50.880	17.200	50.890	25.030	18.350
7	25.440	20.950	10.680	12.150	21.080	18.180	18.390	30.860
Glu								
1	9.370	7.950	16.600	9.060	14.010	8.710	-	-
2	9.960	12.560	6.760	17.320	14.160	2.660	16.410	2.800
3	37.320	8.790	20.880	11.930	10.200	11.270	16.730	8.260
4	4.940	8.690	15.470	5.680	28.790	7.190	4.450	1.620
5	8.040	1.510	8.080	17.210	8.560	17.230	4.300	13.740
6	12.240	13.660	7.640	6.520	3.250	4.130	7.980	15.250
7	38.710	30.190	12.000	7.630	3.970	19.600	12.250	16.460
NAA/Cr								
1	1.190	4.400	2.670	1.470	2.080	1.100	-	-
2	1.420	1.230	1.580	3.200	1.140	1.330	.851	1.790
3	1.460	2.600	5.440	.570	.920	1.770	1.040	1.450
4	13.600	2.540	.590	.128	1.890	2.040	1.390	.597
5	2.130	2.510	1.160	2.160	.460	1.730	2.380	1.340
6	3.080	.503	.839	1.810	2.970	.765	1.030	2.680
7	.787	.683	.516	1.050	.197	.424	2.110	.450
Cho/Cr								
1	1.830	1.180	2.400	2.620	.070	1.940	-	-
2	2.420	.611	3.960	.776	.505	.650	.736	1.130
3	.434	1.730	1.190	.570	2.440	.240	.980	1.110
4	5.720	.765	1.020	.423	2.530	7.370	1.950	.897
5	1.020	4.250	.965	.195	.219	1.990	1.710	1.830
6	2.550	1.760	.491	4.970	2.130	2.040	1.270	2.190
7	1.050	.316	.304	.285	.435	.852	3.060	1.110

WM = white matter, GM = gray matter, NAA = N-acetyl aspartate, Cr = creatine, Cho = choline, Glu = glutamate.

Excluded: Spectra excluded from the final analysis due to substandard spectral quality

Supplementary Table S5. Individual measurements of brain metabolite concentrations and ratios in multi-voxel spectroscopy across four evaluated brain regions (n = 7).

	WM of frontoparietal lobe		GM of frontoparietal lobe		Caudate nucleus		Thalamus	
	Left	Right	Left	Right	Left	Right	Left	Right
NAA								
1	22.730	40.400	21.920	17.300	45.420	29.140	16.360	16.710
2	19.520	17.960	13.010	19.120	22.710	29.470	9.700	14.840
3	-	-	24.650	23.700	21.650	19.950	23.830	19.610
4	40.150	28.130	24.550	29.550	28.320	20.320	11.940	14.260
5	26.680	33.570	46.350	22.690	-	-	28.900	23.010
6	38.070	34.650	13.190	46.250	22.740	28.100	22.640	13.790
7	20.880	21.170	14.320	25.240	-	-	27.150	24.010
Cr								
1	7.750	15.760	12.230	97.390	61.080	22.370	4.510	9.020
2	16.220	6.470	9.030	15.480	12.740	8.160	10.200	6.130
3	-	-	6.850	19.040	20.880	45.160	16.700	16.410
4	8.870	10.010	31.990	33.040	44.260	20.590	10.080	13.510
5	36.690	13.070	22.570	5.330	-	-	21.510	14.270
6	20.920	11.360	18.010	43.760	12.420	13.880	15.310	14.040
7	9.610	9.210	24.900	6.540	-	-	18.800	28.170
Cho								
1	12.040	29.150	9.250	42.220	20.700	14.420	13.080	20.180
2	13.210	24.210	9.370	24.510	15.370	19.470	5.870	9.200
3	-	-	20.270	28.420	28.320	16.070	31.020	12.390
4	25.410	8.580	26.300	8.310	20.410	13.490	12.490	10.380
5	57.620	25.370	60.790	4.540	-	-	9.860	10.520
6	23.440	11.530	14.060	18.430	9.160	8.840	45.680	13.330
7	17.720	11.330	22.410	17.080	-	-	26.820	19.810
Glu								
1	2.130	2.910	5.480	4.970	6.490	5.410	10.260	8.810
2	2.660	10.800	3.030	7.760	1.460	1.040	12.670	6.530
3	-	-	10.250	14.450	7.280	2.110	5.060	5.610
4	0.050	8.810	3.380	15.680	9.340	6.480	6.010	4.230
5	1.260	6.650	5.880	3.600	-	-	7.060	6.190
6	26.740	1.070	1.910	8.930	8.110	10.860	3.140	1.090
7	7.480	3.000	1.990	8.610	-	-	3.080	5.010
NAA/Cr								
1	2.930	2.560	1.790	0.178	0.744	1.300	3.630	1.850
2	1.200	2.780	1.440	1.240	1.780	3.610	4.890	2.420
3	-	-	3.600	1.250	1.040	0.442	1.430	1.190
4	4.530	2.810	0.767	0.894	0.640	0.987	1.180	1.060
5	0.727	2.570	2.050	4.260	-	-	1.340	1.610
6	1.820	3.050	0.732	1.060	1.830	2.020	1.480	0.982
7	2.170	2.300	0.575	3.860	-	-	1.440	0.852
Cho/Cr								
1	1.550	1.850	0.756	0.434	0.339	0.645	2.900	2.240
2	0.815	3.740	1.040	1.580	1.200	2.380	0.570	1.500
3	-	-	2.960	1.490	1.360	0.356	1.860	0.755
4	2.870	0.858	0.822	0.251	0.461	0.655	1.240	0.768
5	1.570	1.940	2.690	0.852	-	-	0.458	0.737
6	1.120	1.020	0.780	0.421	0.737	0.637	2.980	0.949
7	1.840	1.230	0.900	2.610	-	-	1.430	0.703

WM = white matter, GM = gray matter, NAA = N-acetyl aspartate, Cr = creatine, Cho = choline, Glu = glutamate.
Excluded: Spectra excluded from the final analysis due to substandard spectral quality
